# Nursing diagnoses in patients with immune-bullous dermatosis**^1^**

**DOI:** 10.1590/1518-8345.0424.2766

**Published:** 2016-08-15

**Authors:** Euzeli da Silva Brandão, Iraci dos Santos, Regina Serrão Lanzillotti, Adriano Menis Ferreira, Mônica Antar Gamba, Luna Azulay-Abulafia

**Affiliations:** 2 PhD, Adjunct Professor, Escola de Enfermagem Aurora de Afonso Costa, Universidade Federal Fluminense, Niterói, RJ, Brazil.; 3 PhD, Full Professor, Faculdade de Enfermagem, Universidade do Estado do Rio de Janeiro, Rio de Janeiro, RJ, Brazil.; 4 PhD, Full Professor, Instituto de Matemática e Estatística, Universidade do Estado do Rio de Janeiro, Rio de Janeiro, RJ, Brazil.; 5 PhD, Adjunct Professor, Universidade Federal do Mato Grosso do Sul, Três Lagoas, MS, Brazil.; 6 PhD, Associate Professor, Escola Paulista de Enfermagem, Universidade Federal de São Paulo, São Paulo, SP, Brazil.; 7 PhD, Adjunct Professor, Faculdade de Medicina, Universidade do Estado do Rio de Janeiro, Rio de Janeiro, RJ, Brazil.

**Keywords:** Nursing, Nursing Care, Nursing Diagnosis, Dermatology

## Abstract

**Objective:**

identify nursing diagnoses in patients with immune-bullous dermatosis.

**Method:**

a quantitative and descriptive research, carried out in three institutions located in Rio de Janeiro and Mato Grosso do Sul, Brazil, using the Client Assessment Protocol in Dermatology during a nursing consultation. Simple descriptive statistics was used for data analysis.

**Results:**

14 subjects participated in the study, nine with a diagnosis of pemphigus vulgaris, pemphigus two and three of bullous pemphigoid. The age ranged between 27 and 82 years, predominantly females (11). 14 nursing diagnoses were discussed and identified from a clinical rationale in all study participants, representing the most common human responses in this sample. The application of the Assessment Protocol in Dermatology facilitated the comprehensive assessment, in addition to providing the identification of diagnostics according to the North American Nursing Diagnosis Association International.

**Conclusion:**

the nursing diagnoses presented confirm the necessity of interdisciplinary work during the care for this clientele. For better description of the phenomena related to the client in question, it is suggested the inclusion of two risk factors related in three diagnoses of this taxonomy. It is worth noting the contribution of the findings for the care, education and research in nursing in dermatology.

## Introduction

Considering the complexity involving nursing care to clients with immune-bullous dermatosis (ID), just as the lack of theoretical frameworks specifically geared for this care^1^ ^-^ ^2^, the need for the nurse to develop practices and technologies that enable the best care of this clientele, is clear. It is understood that providing nursing care goes beyond the fulfillment of medical prescriptions and thus includes the use of a care technology that involves the evaluation of this client in its entirety and, therefore, interventions that promote comfort and well being. While performing these practices, which also provides autonomy to the nurse, philosophical, theoretical and technological foundation is applied, as supported by the contents described by Henderson^3^. 

Accordingly, the nurse is responsible for the systematization of their care^4^ ^)^ and consequently by the use of the nursing process, a methodological tool that helps to identify, understand, describe, explain and/or predict how the client responds to health problems, determining thus the aspects that require professional intervention. In order that expected results can be achieved, nursing interventions based on the judgment on specific human phenomena, such as, Nursing Diagnoses stand out.^4^_._

The ID are diseases that consist on the development of bubbles and / or blisters on the skin and / or mucous membranes, as a result of activation of the immune system against specific structures of the skin, called autoantigens^5^. These autoantigens can be located in the intra-epidermal or sub-epidermal regions, it is essential to identify their location for classification^5^_._

In the case of pemphigus, the autoantigens are located in the intra-epidermal region, being more frequent in the pemphigus vulgaris and the pemphigus. Among the sub-epidermal ID, the bullous pemphigoid, dermatitis herpetiformis, and acquired epidermolysis bullosa stand out.^5^. Thus, the client developing a ID suddenly has the integrity of their skin compromised, experiencing pain, discomfort, difficulty in moving and resting, which makes it vulnerable to complications, among them, infections, myiasis infestation and dehydration due to the loss of liquids and proteins^2^. 

Although the affected person can present extensive areas of healthy skin, it is noteworthy that all the skin is vulnerable to new lesions arising from any pressure on the skin, even while performing necessary technical procedures, such as blood pressure measurement, the use of a tourniquet, transportation, among others. In addition, there are social and emotional implications on the impossibility of hiding a imprinted skin problem^2^_._

It is worth mentioning the shortage of nursing research on patients with ID, as shown by the results of two integrative literature reviews (ILR)^1^ ^-^ ^2^ which selected, respectively, four and six publications only, featuring case studies or review studies and evidence levels 4 and 5^1^ ^-^ ^2^. In the latest ILR, published in 2013^1^, the four articles were published in English between 2006 and 2009, finding two American authors, a Chinese author and one without identification of authorship. Despite the fact that three articles were published in nursing journals, there was a predominance of medical authors and / or co-authors, and only one of the articles had a nurse as coauthor. 

Despite the studies in Brazil in order to identify nursing diagnoses in people with different problems that affect health, such as diabetes mellitus^6^, sepsis^7^, or subjected to procedures such as prostatectomy^8^ and cardiac surgery^9^, the articles selected during that ILR reveal the inexistence of studies on the nursing diagnoses presented by customers with ID. It was found that the authors of these articles cite nursing care superficially, usually at the end of the articles^1^_,_ emphasizing aspects of the disease and drug therapy^1^ ^-^ ^2^. 

On the use of technology it stands out that, historically, the health care assistance model for health had its focus on hard and soft-hard technologies, for which medical knowledge provide the structure of work for other professionals. The health services resulting from this structure are focused on the prescriptive act, hegemonic and inducive of procedures^10^_._ Despite this history, it is understood that the process of nursing work must have at its care core the hegemony of live labor, ensuring customers comprehensive and integral care^11^_._

Therefore, the importance of the nursing consultation is clear, nursing activity regulated by the Professional Practice Law 7,498 / 86^12^_,_ that gives the nurses autonomy in the field practice to apply their specific knowledge independent from the requirements of other health professionals. Its main focus considers the client's integrity beyond the disease, emphasizing its different dimensions (physical, mental, spiritual)^13^_._

In this perspective there is the possibility of applying the Client Assessment Protocol in Dermatology (PACD - *In Portuguese*), a soft-hard and soft technology, validated by experts in the field^14^_._ Such technologies can be classified into types associated with the work process: the hard one is represented by machines and instruments; the soft-hard related to technical knowledge; and soft technologies are represented by relations^15^_._ The PACD, being a nursing technology, it has relational therapeutic focus with the customers^15^, aiming to enhance their subjectivity, their social background, their family relationships, their values and beliefs, sharing knowledge for self-care. 

Given these considerations, we had as objective to identify nursing diagnoses in clients with immune-bullous dermatosis. In this study, the respective risk factors related and defining features of the diagnoses considered as priorities will be presented from the taxonomy recommended by NANDA *International* (NANDA-I)^16^.

## Methods

Descriptive quantitative research applying the nursing consultation as data production strategy through PACD, in order to identify the nursing diagnoses presented by people with ID. This identification involves the prevalence, incidence, variations and measurable attributes of a phenomenon, as was seen when applying PACD during the nursing consultation. 

Diagnoses, defining characteristics and risk factors were identified in 14 clients hospitalized in the period between June 2012 and April 2013, in three units specialized in dermatology: two located in the university hospitals of Rio de Janeiro and Niteroi, and in a private institution in Campo Grande, Mato Grosso do Sul, Brazil. These institutions were chosen because they own a dermatology ward and because they are a reference for customer service with ID.

The sample was defined by convenience sampling, as the dermatoses in question are considered rare. The sample consisted of clients who met the following inclusion criteria: adults diagnosed with ID, regardless of the history of previous admissions, sex, age and the use or not of systemic medications. Exclusion criteria: people in psychiatric treatment and / or disoriented in time and space.

The data collection instrument denominated Client Assessment Protocol in Dermatology (PACD) was applied to 14 participants by the researcher during admission, ensuring the privacy of the same. The PACD consists of 10 items^14^, including: 1. Identification: name, registration, admission date and birth, address and origin; and socio-demographic variables: gender, age, race / self-reported ethnicity, marital status, education, profession / occupation, family income, nationality, origin and religious belief; 2. History - clinical variables: hypertension, diabetes, kidney disease, allergies, medications, smoking, alcohol use, previous hospitalizations, blood transfusions, previous diseases and family medical history and preventive examinations; 3. Aspects related to skin disease: knowledge regarding the disease, degree of discomfort and emotional and spiritual effects of the illness of the skin; 4. Physiological aspects: walking ability, hearing, vision, fluid intake, nutrients and eliminations; 5. Emotional and social aspects: how the customer sees and feels about the disease; 6. Issues related to hospitalization, in particular the concerns and expectations regarding nursing; 7. Information on physical examination; 8. List of 92 nursing diagnoses, with their defining characteristics and related risk factors, selected from the NANDA-I^16^. The selection of diagnosis was performed considering the specificity of the dermatology clientele, in order to facilitate the identification by nurses; 9. Registration of interventions; 10. Registration of re-evaluations. 

After the evaluation of each customer by applying the PACD, there was a discussion between the researcher and the unit nurses to define the nursing diagnoses.

The PACD was developed by the researcher and validated by the Delphi technique, using a panel of seven judges experts in the dermatology Area who considered its applicability feasible^14^_._ For its use, a software was developed, which is registered (INPI 2325) in the Department of Innovation of the Deputy Dean of Graduate Studies and Research at the State University of Rio de Janeiro (InovUERJ) and co-authored with the Innovation Agency of the office of the Deputy Dean of Research, graduate Studies and Innovation from the Federal University of Fluminense (UFF). 

The analysis of medical records of 14 participants in the survey was conducted to complement information, considering questions arising from the admission. To evaluate the data, simple descriptive statistics were used.

The thesis project that originated this proposal was submitted to the Ethics Committee at the Hospital Universitário Pedro Ernesto UERJ and approved in conformity with the protocol 0258.0.228.000-11 . The development of the study met the national and international standards of ethics in research involving human subjects, following the Resolution 466/2012^17^_._

## Results

**Figure 1:**
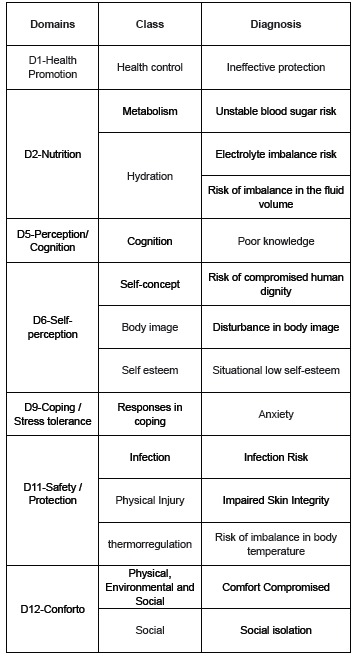
Domains, classes and their respecting nursing diagnoses found in 14 study participants. Niteroi, RJ and Campo Grande, MS, Brazil, 2012-2013.

14 clients participated in the study: Nine with a diagnosis of pemphigus vulgaris, two pemphigus and three with bullous pemphigoid. The age ranged between 27 and 82 years, predominantly 11 females. Figure 1 shows the domains and classes related to the 14 nursing diagnoses presented by the participants.

Constituting a significant number of nursing diagnoses identified, the defining characteristics and risk factors related to six diagnoses are described as follows, being considered to be a priority on the clinical situation and vulnerability to complications presented by customers.

## Discussion

The nursing diagnosis "ineffective protection" reflects the vulnerability of clients with ID to physical and biological risks, justified by the fragility of the skin, extensive pre-existing skin lesions, and the use of corticosteroids and / or immunosuppressants in high doses^5^. On the fragility of the skin, it is emphasized that any pressure in apparently normal skin, especially near the injury induces epidermal detachment. Although the individual may present large areas of healthy skin, it is noteworthy that all the skin is vulnerable to further injury. Thus, any pressure on the skin, even during routine nursing care, can increase the injured area, and this a fact confirmed by the positive Nikolski sign (+). This signal is characterized by partial or complete detachment of the epidermis, which is made through the finger pressure on perilesional skin^5^.

On the extent of injuries in the case of the nine clients with a diagnosis of pemphigus vulgaris, it was possible to perform evaluation by the Penfigo Vulgar's mucuous cutaneous compromised skin index^18^. It was found that three participants had a index of 60, followed by two with index equal to 80. The other four had equal rates of 30, 35, 40 and 100. Given this assessment, it can be said that of the nine patients with pemphigus vulgaris, six had a very significant compromised mucous cutaneous index, ie above 60. Subjects with other immune bullous dermatosis were not measured in this index, since this is index is designed specifically for those with pemphigus vulgaris^18^.

Prednisone is usually given at high doses. The prescribed initial daily dose is 1 to 2mg/kg, depending on the severity of the disease^5^. In the case of this research, the daily doses were between 60 to 120mg/day. After a week, with no improvement with corticosteroid therapy alone, it is indicated an immunosuppressant drug, the main one being azathioprine at a dose of 2mg/day^5^. It is noteworthy that during treatment, many customers can develop complications^5^ ^)^ such as hypertension, hyperglycemia, besides having greater vulnerability to infection and subsequent sepsis, which can lead them to death. This fact should be a warning to nurses about the importance of their intervention to control and reduce the vulnerability of clients to risk.

The "impaired skin integrity" was found before the clinical manifestation of primary ID, which is the development of bubbles and / or blisters on the skin and / or mucous^5^. Disruption of bullous lesions gives rise to eroded, exulcerated and exudative lesions, with possible loss of fluid and protein, a group of symptoms that predisposes to infection, dehydration, anemia and deep malnutrition. Given this fact, it is suggested the inclusion of the related factor (internal) "immune bullous dermatosis" in this nursing diagnosis, favoring the identification and the best description of this diagnosis of NANDA-I particularly in the dermatology field. 

The "risk of infection", defined by the risk of being invaded by pathogenic organisms^16^, becomes a phenomenon to be monitored due to the compromised skin integrity and high doses of steroids used in the disease treatment^5^. Customers are more vulnerable in the hospital, especially those with advanced age, diabetes, and other factors. There is also the absence of curative, which enhances the vulnerability to biological agents^1^ ^-^ ^2^_._ It is noteworthy the problems faced by health professionals that care for this clientele, highlighting working conditions resulting from pauperization of health services in the public system. 

Facing the interference of skin condition in the physical, mental, environmental and social spheres makes the identification of the diagnosis "impaired comfort" understandable. Regarding this diagnosis, there is the pain caused by lack of skin integrity and the difficulty presented by the customer to rest in bed, both interfering negatively in sleep patterns. Moreover, it is necessary to mention the discomfort caused by the customer's exposure to the stigmatizing eyes of society, given the impossibility of hiding a problem that is imprinted on your skin. These factors make the evaluation of that client in their entirety essential in order to identify the problems and implementation of nursing actions that promote comfort^11^_._

Regarding the diagnosis "Risk of compromised human dignity", there is the inevitable exposure of the lesions, which prevents a person's right to omit a health problem that is imprinted on the skin. Mistaken belief about the possibility of contagion can derail social relations, the continuation of studies, work and, in some cases, even family life^13^.

The presence of the nursing diagnosis "lack of knowledge" was predictable, because they are uncommon diseases not disclosed in the media, which hinders the understanding of the disease process, treatment adherence and self-care. This fact confirms the importance of clarifying customer questions^14^ ^)^ aiming the preparation for hospital discharge. However, it is important to point out that during the aggravation phase of the disease, the guidelines often become irrelevant because the pain and discomfort did not allow assimilation of them by the customer. In this phase, the guidelines should be carried out according to the demand of the subject itself. Guidelines related to the etiology of the disease and the possibility to control it with treatment can reduce anxiety and improve coping responses.

Although not considered a priority, the diagnosis "unstable glycaemia risk", "Electrolyte imbalance risk" and "Risk of imbalance in the fluid volume" deserve some comments, especially because the customers make use of corticosteroids in high doses, according to the participants in this study.

Regarding the "unstable glycaemia risk," alert to the nurse responsibility for customer guidance for self-care, in view of the presence of the risk factor "lack of knowledge about diabetes control," including those that show changes in glucose level before starting treatment. Given the absence in NANDA-I^16^ of a risk factor that specify the use of this drug, we suggest the inclusion of the risk factor "corticosteroids". It is believed that this can assist nurses in identifying the diagnosis "unstable glycaemia risk", not only for those working in the dermatology field, but also in other areas, given that corticosteroids are used to treat various diseases different specialties. 

On the diagnostic "Electrolyte imbalance risk" and "risk of imbalance in the volume of fluid", these are usually present in this clientele due to loss of electrolytes in the cases of large eroded and/or bullous exudative areas^5^ ^)^ and consequent fluid retention with the use of corticosteroids. In the specific cases of customers with pemphigus vulgaris, which in addition to the skin lesions have lesions in the oral mucosa, the risk is increased due to the difficulty of ingesting liquids. Consequently, reduction can occur, increasing or rapidly changing from one location to another intravascular, interstitial and / or intracellular fluid, setting the diagnosis "risk of imbalance in the volume of fluid"^16^_._ In the absence of risk factors such as "extensive skin lesions" and "use of corticosteroids," which can cause loss or excessive fluid retention, inclusion is recommended in NANDA-I^16^.

The small number of participants in this research is justified because it is a group of uncommon diseases^5^, thus being one of the limitations of this study. 

**Figure 2:**
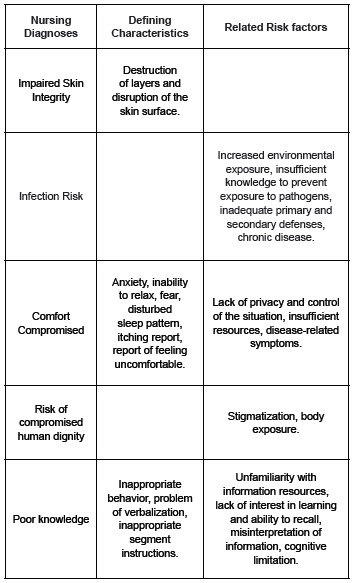
Nursing diagnoses considered a priority in the 14 study participants, with their defining characteristics and risk factors / related. Niteroi, RJ and Campo Grande, MS, Brazil, 2012-2013.

These diagnoses translated the complexity of customers and hence the importance of nursing care needed for their recovery. On this, it is emphasized that the diagnosis considered a priority, as shown in Figure 2, were invaluable for the implementation of essential nursing interventions. The description of the interventions needs to be the next step in order to promote the comfort and injury prevention, essential for the recovery of these customers.

## Conclusion

The study identified 14 nursing diagnoses representative of the needs of 14 customers affected by ID through comprehensive and personalized assessment, favored by the implementation of the PACD. Of these, six were considered a priority. We emphasize the importance of using this protocol / technology, considering that it facilitated the identification of diagnostics, thus revealing a useful tool, easy to use and that provides assessment of this client in its entirety. 

Two risk factors or related could be added in three diagnoses of NANDA-I Taxonomy for better description of the phenomena when related to the clientele in question: 1. "Use of corticosteroids" (diagnosis "unstable glycaemia risk" and "risk of imbalance in the volume of fluid"); 2. "extensive skin lesions" (diagnosis "risk of electrolyte imbalance" and "risk of imbalance in the volume of fluid"). It is suggested to carry out further studies and the inclusion of these factors in NANDA-I, to facilitate the use of diagnostics in everyday practice.

Given the incipient nature of nursing studies toward the client with DI, we consider this work a contribution to inspire other researchers and make available to specialist nurses and / or general practitioners information on the problems presented by this clientele, envisioning personalized nursing care, aiming to the systematizing and contribution to the care, education, and research of nursing in the dermatology field.
